# Correlation between intestinal flora characteristics and immune function in COPD patients treated with non-invasive ventilator and its value in predicting ventilator-associated pneumonia: A prospective study

**DOI:** 10.1097/MD.0000000000049275

**Published:** 2026-06-12

**Authors:** Yanbo Liu, Chengjiang Liu, Caifeng Huang, Lisha Pang

**Affiliations:** a Department of Emergency, Sir Run Run Shaw Hospital, Zhejiang University School of Medicine, Hangzhou, Zhejiang, China; b Department of General Medicine, Affiliated Anqing First People’s Hospital of Anhui Medical University, Anqing, Anhui, China; c Department of Critical Care Medicine, Tongde Hospital of Zhejiang Province Affiliated to Zhejiang Chinese Medical University (Tongde Hospital of Zhejiang Province), Hangzhou, Zhejiang, China.

**Keywords:** chronic obstructive pulmonary disease, immune function, intestinal flora, noninvasive mechanical ventilation, ventilator-associated pneumonia

## Abstract

To explore the relationship between intestinal flora characteristics and immune function in patients with chronic obstructive pulmonary disease (COPD) treated with noninvasive ventilator (NIV), and to analyze the factors affecting the occurrence of ventilator-associated pneumonia (VAP) in patients. A prospective study analyzed 280 COPD patients on noninvasive ventilation from August 2023 to August 2024, divided into VAP group and non-VAP group based on 48-hour VAP occurrence. The study explored links between gut microbiota and immune function and utilized binary logistic regression and a random forest model to predict VAP risk. A correlation was observed between intestinal flora characteristics and immune features in COPD patients, revealing complex interactions between bacterial subgroups and immune cell levels. Re-intubation, mechanical ventilation time ≥ 4 days, smoking, Escherichia coli, Enterococcus faecium, and Enterococcus faecalis were risk factors for VAP in COPD patients (OR* *= 2.800, 3.079, 4.665, 1.781, 1.342, and 1.600, all *P *< .05). In addition, Bifidobacterium, Lactobacillus, CD3^+^, CD4^+^, and CD8^+^ were protective factors (OR* *= 0.557, 0.801, 0.534, 0.349, and 0.134, all *P *< .05). Among these, the top 3 important factors were Lactobacillus, Enterococcus faecalis, and CD3^+^, (%IncMse × 10^–2^: 37.962%, 31.552%, 29.141%, respectively). The random forest model demonstrated significant predictive capability (*P *= .01, *r*^2^ =0.538); with the highest diagnostic performance under 10 factors (area under the curve = 0.908), including flora and immune characteristics. In addition, the area under the curve of the random forest model for predicting the occurrence of VAP in COPD patients was 0.857. This study preliminarily suggests that the characteristics of intestinal flora in COPD patients treated with NIV may be associated with immune function and may be involved in the occurrence of VAP. The random forest model based on intestinal flora has shown preliminary predictive value in predicting the occurrence of VAP, but more large-scale and multi-center studies are needed to further verify its clinical applicability.

## 1. Introduction

Chronic obstructive pulmonary disease (COPD) is a common, preventable, and treatable chronic airway disease. It is caused by chronic airflow obstruction due to airway disease or lung parenchymal destruction. The main clinical features are persistent airflow limitation and corresponding respiratory symptoms, such as dyspnea, cough, and airflow obstruction.^[1]^ COPD can cause permanent damage to the respiratory tract and lungs, further aggravating the patient’s clinical symptoms, seriously affecting the patient’s quality of life, and even threatening the patient’s life safety.^[2]^ According to *The China Pulmonary Health Study* led by Academician Wang Chen,^[3]^ the incidence of COPD in adults over 20 years old in China is 8.6%, and it is as high as 13.7% in people over 40 years old, with an estimated patient population of nearly 100 million COPD patients in China. Furthermore, the Global Burden of Disease Study ranks COPD as the fifth leading cause of death in China, highlighting its significance as a major public health issue and a key target in the *Healthy China 2030 Action Plan*.^[4]^

COPD is categorized into acute exacerbation and stable phases. Acute exacerbation is mainly manifested by worsening respiratory symptoms, which is the main cause of death. Clinically, it often necessitates adjustments in treatment plans based on the patient’s condition.^[5]^ The main treatment methods for patients with acute exacerbation of COPD are airway dilation and respiratory support to maintain normal oxygen supply to the body and improve the patient’s clinical manifestations. noninvasive mechanical ventilation (NIV) is a type of mechanical ventilation that provides ventilation through the patient’s upper respiratory tract through the nose or nasal mask. While ensuring the body’s oxygen supply, it reduces breathing work and improves the patient’s lung mechanics.^[6]^ However, with the widespread use of ventilators, the incidence of ventilator-associated pneumonia (VAP) has also increased. According to data from the relevant guidelines in 2018,^[7]^ the incidence of VAP during mechanical ventilation is 9.7 % to 48.4%, and the mortality rate is 21.2% to 43.2%. VAP has become a difficult problem in the treatment of patients undergoing mechanical ventilation. Adopting a preventive approach, it is crucial to identify factors contributing to VAP occurrence in ventilated patients and implement targeted interventions to reduce its risk.

Wang et al ^[8]^proposed the existence of an intrinsic connection between the respiratory tract and the intestine via the “gut-lung” axis. Both the respiratory tract and the gut are hollow organs directly exposed to the external environment, sharing a common embryonic origin. Changes in the intestinal flora may affect respiratory function through the “gut-lung” axis. The intestinal flora is a complex ecosystem. There are about 40 trillion microorganisms in the human intestine. Currently, over 1000 bacterial species have been identified, with 30 to 40 being most common, such as Bifidobacterium, Lactobacillus, and Escherichia coli. Microorganisms colonizing the intestine and respiratory tract can directly regulate tissue function. Intestinal microorganisms can migrate to the lungs and accumulate, thereby inducing infection.^[9]^ As the largest immune organ in the human body, the intestinal flora plays an important role in maintaining the body’s normal immune function. There are reports that probiotic preparations can affect the body’s immune function and enhance its anti-infection ability by regulating the intestinal flora.^[10]^ This shows that the intestinal flora may be involved in the occurrence and development of VAP. To further clarify the role of the intestinal flora in the occurrence of VAP in COPD patients treated with noninvasive ventilator, this study observed the characteristics of the intestinal flora in patients with acute COPD, analyzed its correlation with the patients’ immune function, and constructed a prediction model to provide new ideas for the clinical prevention of VAP.

## 2. Materials and methods

### 2.1. General data

A prospective study was conducted, and 280 COPD patients who underwent noninvasive mechanical ventilation were selected as the study subjects from August 2023 to August 2024. The patients had signed the informed consent.

### 2.2. Inclusion criteria

Inclusion criteria: COPD meets the diagnostic criteria of the *Guidelines for the diagnosis and management of chronic obstructive pulmonary disease (revised version 2021*)^[11]^; Age ≥ 18 years; In acute exacerbation phase; Meet the indications for noninvasive mechanical ventilation; Mechanical ventilation time ≥ 48 hours. Exclusion criteria: Long-term immunosuppressive therapy; Combined with acute respiratory distress syndrome; Combined with blood system diseases; Intestinal function failure; Patients had taken antibiotics within 7 days after admission; Combined with other respiratory diseases; Previous history of mental illness.

### 2.3. VAP diagnostic criteria and grouping method

Patients were referred to the criteria in the *Guidelines for the Diagnosis and Treatment of Hospital-Acquired Pneumonia and Ventilator-Associated Pneumonia in Adults in China (2018 Edition*)^[7]^Chest X-ray or CT showed new or progressive infiltration, consolidation or ground-glass opacity, accompanied by 2 or more of the following 3 clinical symptoms: fever (body temperature > 38°C); purulent airway secretions; peripheral white blood cell count > 10 × 10^9^/L or < 4 × 10^9^/L. Note: The diagnostic criteria of VAP was originally formulated for patients with invasive mechanical ventilation, and this study applied it to patients with noninvasive ventilation, because both of them have the risk of respiratory tract infection and similar clinical signs. At present, the diagnostic criteria of noninvasive ventilation associated pneumonia are not unified, and most studies use similar VAP criteria, so this study refers to the criteria for diagnosis.

### 2.4. Intestinal flora detection method

On the second day of admission, 0.5g of fresh stool was collected in the morning and processed within 30 minutes. The sample was diluted to a 10^−8^ concentration using a 10-fold dilution method. Then 10 μl was cultured in anaerobic culture medium (Remel, Inc., Device Registration No. 20152402951) for lactobacillus and bifidobacterium, and in aerobic culture medium (Remel, Inc., Device Registration No. 20152402273) for Escherichia coli, Enterococcus faecalis, and Enterococcus faecalis. The culture medium was placed in the fully automatic microbial culture system BacT/ALERT® VIRTUO® (bioMerieux, Inc., Device Registration No. 20172221556) and set at 37°C for 48 hours. After 48 hours, a fully automatic microbial identification and drug sensitivity analyzer (bioMerieux, Inc., Device Registration No. 20172221402) was used to identify and count colonies, and the average number of counted colonies was expressed as the logarithm of colony-forming units per gram of wet stool (lg CFU/g). The selective culture method in this study is based on the following considerations: it is consistent with the routine process of clinical microbiology laboratory, which is convenient for the transformation of results; it focuses on culturable potential pathogenic bacteria (such as Enterobacteriaceae, Enterococcus) and their drug resistance phenotypes, which is consistent with the scientific hypothesis of this study to explore the translocation of pathogenic bacteria in the “gut-lung axis”; to ensure the feasibility and repeatability of sample processing under the condition of limited resources.

### 2.5. T lymphocyte subset detection method

On the second day of admission, 5 mL of fasting venous blood was collected from the patient, and the CD3^+^, CD4^+^, and CD8^+^ levels were detected using a BD FACSLyric flow cytometer (BD Biosciences, Device Registration No. 20192220383).

### 2.6. Pulmonary function test method

On the second day of admission, pulmonary function tests were performed using the VyntusSpirometry Family device (Vyaire Medical GmbH, Device Registration No. 20242070524). Measurements included forced expiratory volume in 1 second (FEV1), forced vital capacity (FVC), and diffusing capacity of the lungs for carbon monoxide (DLCO).

### 2.7. Detection method of inflammatory factors

On the second day of admission, 5 mL of fasting venous blood was collected from the patient and centrifuged at 3200 r/min for 10 minutes (radius 12 cm). Serum levels of tumor necrosis factor-α (TNF-α) and interleukin-6 (IL-6) were detected using the fully automatic biochemical analyzer cobas pure c 303 (Roche Diagnostics GmbH, Device Registration No. 20232220473).

### 2.8. Method for assessment of disease severity

On the second day of admission, the COPD assessment test (CAT) scale was used to assess the severity of the patient’s disease. There are 8 items in total, each with a score of 0 to 5. The higher the score, the more severe the patient’s disease.

### 2.9. Statistical method for baseline data

The hospital pathology system was used to collect the baseline data of patients, including gender, age, body mass index (BMI), disease course, pre-hospital exacerbation duration, reintubation, mechanical ventilation time ≥ 4 days, comorbidities (e.g., hyperlipidemia, hypertension, diabetes, coronary heart disease), smoking, and drinking.

### 2.10. Safety precautions

Personnel safety measures: Laboratory staff should wear clothing appropriate for laboratory work and safety equipment such as gloves and goggles. In the work area, comfortable, non-slip footwear that protects the feet should be worn. Laboratory personnel must wash their hands immediately after removing gloves, before leaving the laboratory, and before eating. After handling cultures, hands should be washed promptly. Sample Collection Safety Measures: Staff should wear masks and disposable gloves. A small portion of the fecal sample should be collected using a fecal swab and immediately placed into a sterile container. The sample must be transported to the laboratory within 30 minutes of collection. Sample Collection Precautions: Patients should avoid using antibiotics for 3 days before sample collection, and female patients should avoid collection during menstruation. Staff must ensure their hands are clean before sample collection and should not use antibacterial soap or hand sanitizer. Disposable gloves should be worn, and care must be taken to avoid contact with urine, menstrual blood, or toilet surfaces during the collection process. Final Sample Disposal: Laboratory samples and waste generated during testing should be placed in appropriate containers and leak-proof autoclave bags. At the end of the experiment, all waste must be sterilized onsite by autoclaving at 121 °C for 30 minutes, then transported to a designated facility for incineration. All experimental procedures (including flora detection and immune index determination) were carried out in accordance with the Clinical Microbiology Operating Standards (2021 edition), and the sampling time point, instrument parameters and quality control measures were described in detail in the method section. Statistical analysis was performed using SPSS 26.0 software, and parameter settings (e.g., number of trees, number of cross-validations) for model construction (e.g., random forest) were provided in the supplementary material to ensure peer reproducibility.

### 2.11. Statistical methods

SPSS25.0 software was used for data processing. The measurement data were expressed as “mean** ± **sd.” The independent sample t test was used for comparison between the 2 groups. The count data were expressed as n% and the χ^2^ test was used. The bivariate Pearson correlation was used to analyze the relationship between the characteristics of the intestinal flora (Bifidobacterium, Escherichia coli, Lactobacillus, Enterococcus faecium, Enterococcus faecalis) and their immune function [T lymphocyte subsets (CD3^+^, CD4^+^, CD8^+^)] of patients. Binary logistic regression was used to analyze the relationship between the characteristics of the intestinal flora of COPD patients and the occurrence of VAP. The random forest model was constructed using the random Forest package in R (R4.1.0) and was internally validated using Bootstrap resampling. The receiver operating characteristic curve was drawn and the area under the curve (AUC) was calculated to test the predictive value of the random forest model for the occurrence of VAP in COPD patients treated with noninvasive ventilator. The significance level was set at α = 0.05.

## 3. Results

### 3.1. Levels of intestinal flora, immune function, lung function, inflammatory factors, and CAT score in COPD patients

**Figure 1.**
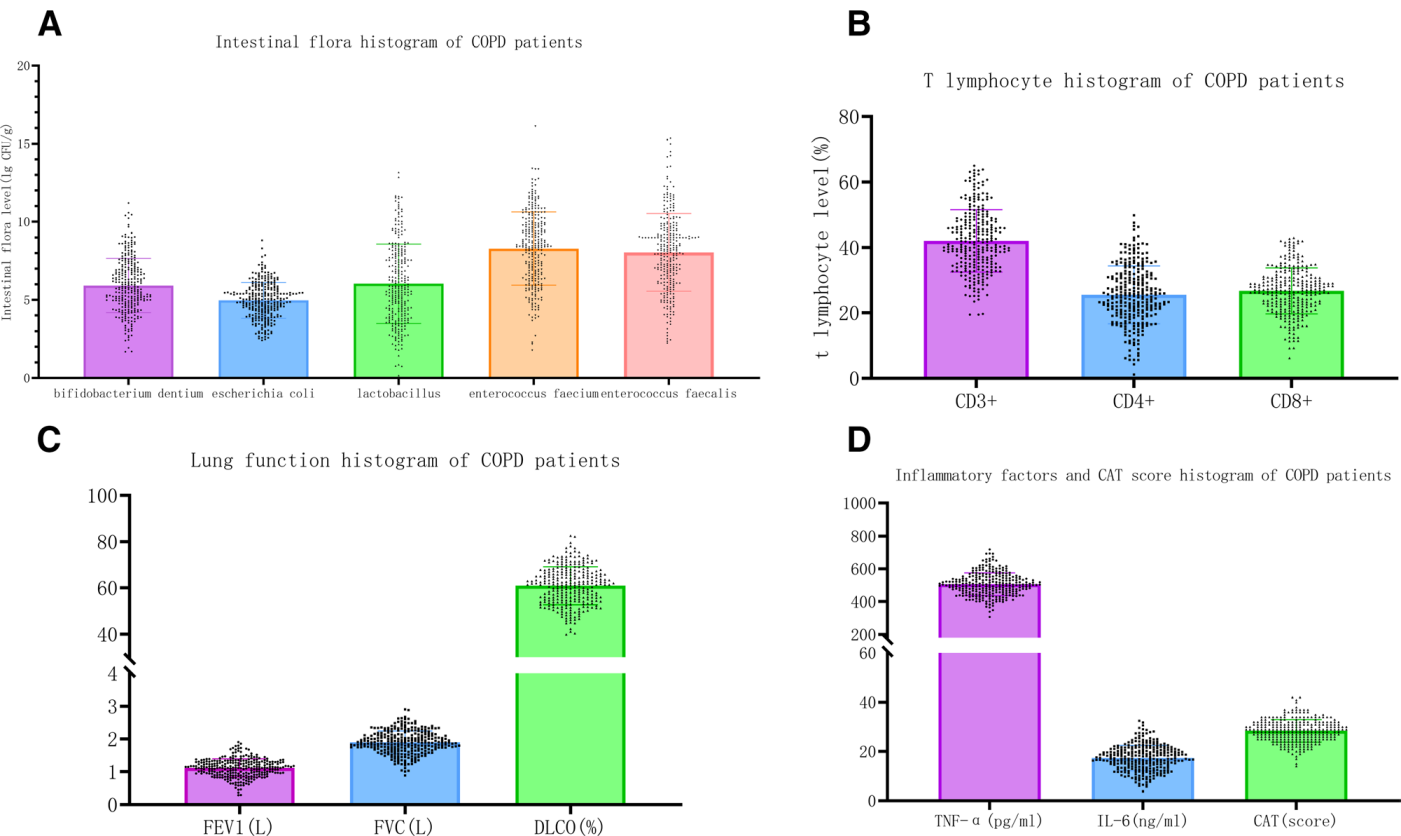
Bar chart of immune function, lung function, inflammatory factors and CAT score of COPD patients. (A) Intestinal flora histogram of COPD patients. (B) T lymphocyte histogram of COPD patients. (C) Lung function histogram of COPD patients. (D) Inflammatory factors and CAT score histogram of COPD patients. CAT = COPD assessment test, COPD = chronic obstructive pulmonary disease.

As shown in Figure 1A, the levels of Bifidobacterium (5.92 ± 1.74) lg CFU/g, Escherichia coli (4.97 ± 1.15) lg CFU/g, Lactobacillus (6.01 ± 2.55) lg CFU/g, Enterococcus faecium (8.29 ± 2.34) lg CFU/g, and Enterococcus faecalis (8.04 ± 2.48) lg CFU/g in COPD patients were high. As shown in Figure 1B, the levels of T lymphocytes in COPD patients were CD3^+^ (42.05 ± 9.51) %, CD4^+^ (25.55 ± 8.86) %, and CD8 ^+^ (26.76 ± 7.05) %, respectively, of which CD3^+^ and CD4^+^ were at low levels compared with normal values. As shown in Figure 1C, the lung function indicators of COPD patients were FEV1 (1.12 ± 0.78) L, FVC (1.90 ± 0.35) L, and DLCO (60.98 ± 8.20) %, respectively, indicating that COPD patients had poor lung function. Additionally, as shown in Figure 1C, the inflammatory factors and disease scores of COPD patients were TNF-α (505.57 ± 69.37) pg/mL, IL-6 (17.35 ± 5.20) ng/mL, and CAT score (28.41 ± 4.60), respectively, indicating that COPD patients had significant inflammatory reactions and were in a more serious condition.

### 3.2. Correlation analysis between intestinal flora levels and immune function, lung function, inflammatory factors, and CAT scores in COPD patients

**Figure 2.**
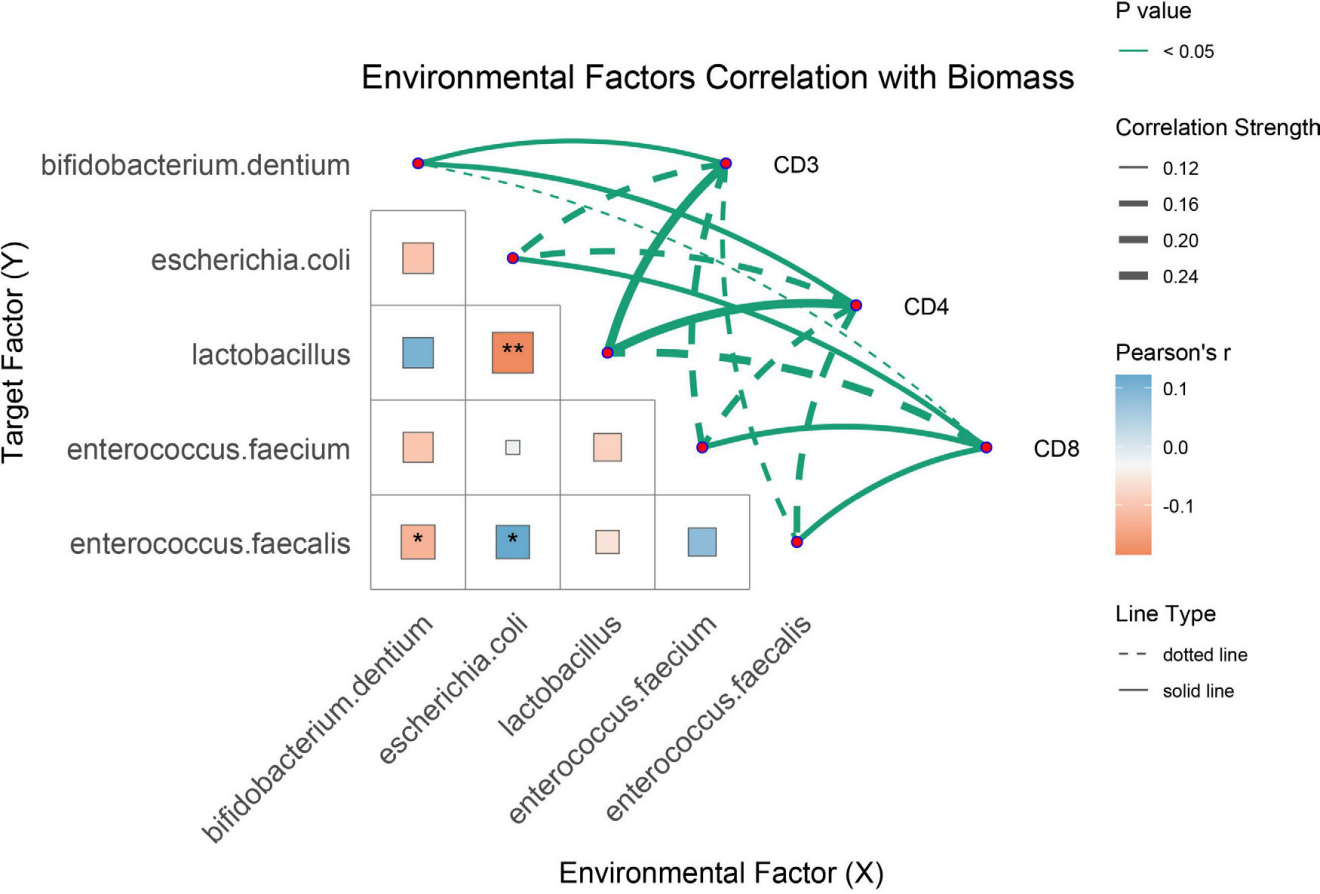
Correlation between intestinal flora levels and immune function, lung function, inflammatory factors and CAT scores in COPD patients. CAT = COPD assessment test, COPD = chronic obstructive pulmonary disease.

As shown in Figure 2, bifidobacteria in COPD patients were positively correlated with CD3^+^ and CD4^+^ (*r *= 0.139, 0.146, both *P *< .05), and negatively correlated with CD8^+^ (*r = *−0.119, *P < *.05); Escherichia coli was negatively correlated with CD3^+^ and CD4^+^ (*r = *−0.169, −0.174, both *P < *.05), and positively correlated with CD8^+^ and CAT score (*r *= 0.146, 0.170, both *P < *.05); the level of Lactobacillus was positively correlated with CD3^+^ and CD4^+^ (*r* = 0.227, 0.211, both *P < *.05), and negatively correlated with CD8^+^ and CAT score (*r *= −0.179, −0.144, both *P < *.05); Enterococcus faecium was negatively correlated with CD3^+^ and CD4^+^ (*r = *−0.176, −0.175, both *P < *.05), and positively correlated with CD8^+^ (*r* = 0.155, *P*<.05); Enterococcus faecalis was negatively correlated with CD3^+^, CD4^+^, and DLCO (*r = *−0.144, −0.179, −0.153, all *P*<.05), and positively correlated with CD8^+^ (*r *= 0.155, *P*<.05).

### 3.3. Comparison of baseline data between VAP and non-VAP groups of COPD patients

A total of 280 COPD patients were included in this study. According to the occurrence of VAP during ventilation, they were divided into VAP group and non-VAP group. The gender, age, body mass index, disease course, pre-hospital exacerbation duration, reintubation, mechanical ventilation time ≥ 4 days, comorbidities, smoking, drinking, FEV1, FVC, DLCO, TNF-α, IL-6, CAT score, levels of Bifidobacterium, Escherichia coli, Lactobacillus, Enterococcus faecium, Enterococcus faecalis, and CD3^+^, CD4^+^, CD8^+^ were analyzed and compared. As shown in Table 1, the proportion of patients in the VAP group who were reintubated, had mechanical ventilation time ≥ 4 days, and smoked [41.13% (51/124), 66.94% (83/124), 66.13% (82/124)] were higher than that of non-VAP [24.36% (38/156), 46.15% (72/156), 44.23% (69/156)]. In addition, the FEV, Bifidobacterium, Lactobacillus, CD3+, CD4^+^ levels in the VAP group [(1.08 ± 0.27)L, (5.22 ± 1.43)lg CFU/g, (5.17 ± 1.78)lg CFU/g, (38.15 ± 8.25)%, (45.16 ± 9.33)%] were lower than those in the non-VAP group [(1.15 ± 0.28)L, (6.48 ± 1.77)lg CFU/g, (6.79 ± 2.83)lg CFU/g, (22.02 ± 7.18)%, (28.35 ± 9.09)%], while TNF-α, CAT score, Escherichia coli, Enterococcus faecalis, Enterococcus faecalis, CD8 ^+^ [(516.08 ± 76.33) pg/ml, (29.05 ± 4.47) points, (5.39 ± 1.12) lg CFU/g, (9.05 ± 2.34) lg CFU/g, (7.69 ± 2.18) lg CFU/g, (29.07 ± 6.23)%] were higher than those in the non-VAP group [(497.23 ± 62.29) pg/ml, (27.91 ± 4.66) points, (4.62 ± 1.04) lg CFU/g, (8.99 ± 2.32) lg CFU/g, (7.29 ± 2.34) lg CFU/g, (24.92 ± 7.15)%] (*P* <.05).

**Table 1:** Comparison of baseline data of COPD patients between VAP group and non-VAP group.

Characteristic		VAP group (n = 124)	Non-VAP group (n = 156)	Statistics	*P*
Gender	Male	79 (63.71)	93 (59.62)	0.489	.485
	Female	45 (36.29)	63 (40.38)		
Age	≥ 60 years	88 (70.97)	101 (64.74)	1.220	.269
	<60 years old	36 (29.03)	55 (35.26)		
BMI (kg/m^2^)		24.09 ± 1.52	24.31 ± 1.69	1.131	.259
Disease duration (years)		8.15 ± 2.98	7.69 ± 2.75	1.340	.182
Duration of Pre-hospital Exacerbation (d)		6.26 ± 2.19	5.92 ± 1.67	1.474	.142
Reintubation	Yes	51 (41.13)	38 (24.36)	8.961	.003
	No	73 (58.87)	118 (75.64)		
Mechanical ventilation ≥ 4 days	Yes	83 (66.94)	72 (46.15)	12.073	<.001
	No	41 (33.06)	84 (53.85)		
Comorbidities	Hyperlipidemia	73 (58.87)	79 (50.64)	1.886	.170
	Hypertension	82 (66.13)	90 (57.69)	2.075	.150
	Diabetes	31 (25.00)	35 (22.44)	0.252	.616
	Coronary heart disease	46 (37.10)	49 (31.41)	0.997	.318
Smoking	Yes	82 (66.13)	69 (44.23)	13.334	<.001
	No	42 (33.87)	87 (55.77)		
Drinking	Yes	88 (70.97)	97 (62.18)	2.380	.123
	No	36 (29.03)	59 (37.82)		
FEV1 (L)		1.08 ± 0.27	1.15 ± 0.28	2.130	.034
FVC (L)		1.875 ± 0.31	1.92 ± 0.38	1.010	.314
DLCO (%)		60.44 ± 8.17	61.41 ± 8.22	1.032	.303
TNF-α (pg/ml)		516.08 ± 76.33	497.23 ± 62.29	2.275	.024
IL-6 (ng/ml)		17.77 ± 5.29	17.02 ± 5.11	1.201	.231
CAT score		29.05 ± 4.47	27.91 ± 4.66	2.067	.040
Bifidobacterium (lg CFU/g)		5.22 ± 1.44	6.48 ± 1.77	6.449	<.001
Escherichia coli (lg CFU/g)		5.39 ± 1.13	4.63 ± 1.05	5.919	<.001
Lactobacillus (lg CFU/g)		5.03 ± 1.72	6.79 ± 2.83	6.086	<.001
Enterococcus faecium (lg CFU/g)		9.05 ± 2.34	7.69 ± 2.18	5.30	<.001
Enterococcus faecalis (lg CFU/g)		9.00 ± 2.33	7.29 ± 2.35	6.084	<.001
CD3^+^ (%)		38.15 ± 8.25	45.5 ± 9.33	6.574	<.001
CD4^+^ (%)		22.03 ± 7.19	28.35 ± 9.09	6.330	<.001
CD8^+^ (%)		29.07 ± 6.24	24.93 ± 7.15	5.105	<.001

BMI = body mass index, CAT = COPD assessment test, COPD = chronic obstructive pulmonary disease, DLCO = diffusing capacity of the lungs for carbon monoxide, FEV1 = forced expiratory volume in 1 second, FVC = forced vital capacity, NIV = noninvasive ventilator, VAP = ventilator-associated pneumonia.

### 3.4. Binary logistic regression analysis of factors affecting the occurrence of VAP in COPD patients

The occurrence of VAP in COPD patients was set as the dependent variable (“1” = VAP group, “0” = non-VAP group), and the data with statistically significant differences in Table 1 (mechanical ventilation time ≥ 4 days, smoking, Bifidobacterium, Escherichia coli, Lactobacillus, Enterococcus faecium, Enterococcus faecalis, CD3^+^, CD4^+^, CD8^+^) were used as independent variables (assignment instructions: mechanical ventilation time ≥ 4 days, smoking: “1” = yes, “0” = no, and the rest of the variables are continuous variables). Table 1 shows that reintubation, mechanical ventilation time ≥ 4 days, smoking, *Escherichia coli, Enterococcus faecium*, and *Enterococcus faecalis* are risk factors for VAP in COPD patients (odds ratio [OR]* *= 2.800, 3.079, 4.665, 1.781, 1.342, 1.600, all *P < *.05). In addition, Bifidobacterium, Lactobacillus, CD3^+^, CD4^+^, and CD8^+^ are protective factors (OR* *= 0.557, 0.801, 0.534, 0.349, 0.134, all *P*<.05). In this study, adjustments have been made for potential confounding factors, including age and BMI.

### 3.5. Construction of a random forest model affecting the occurrence of VAP in COPD patients

**Figure 3.**
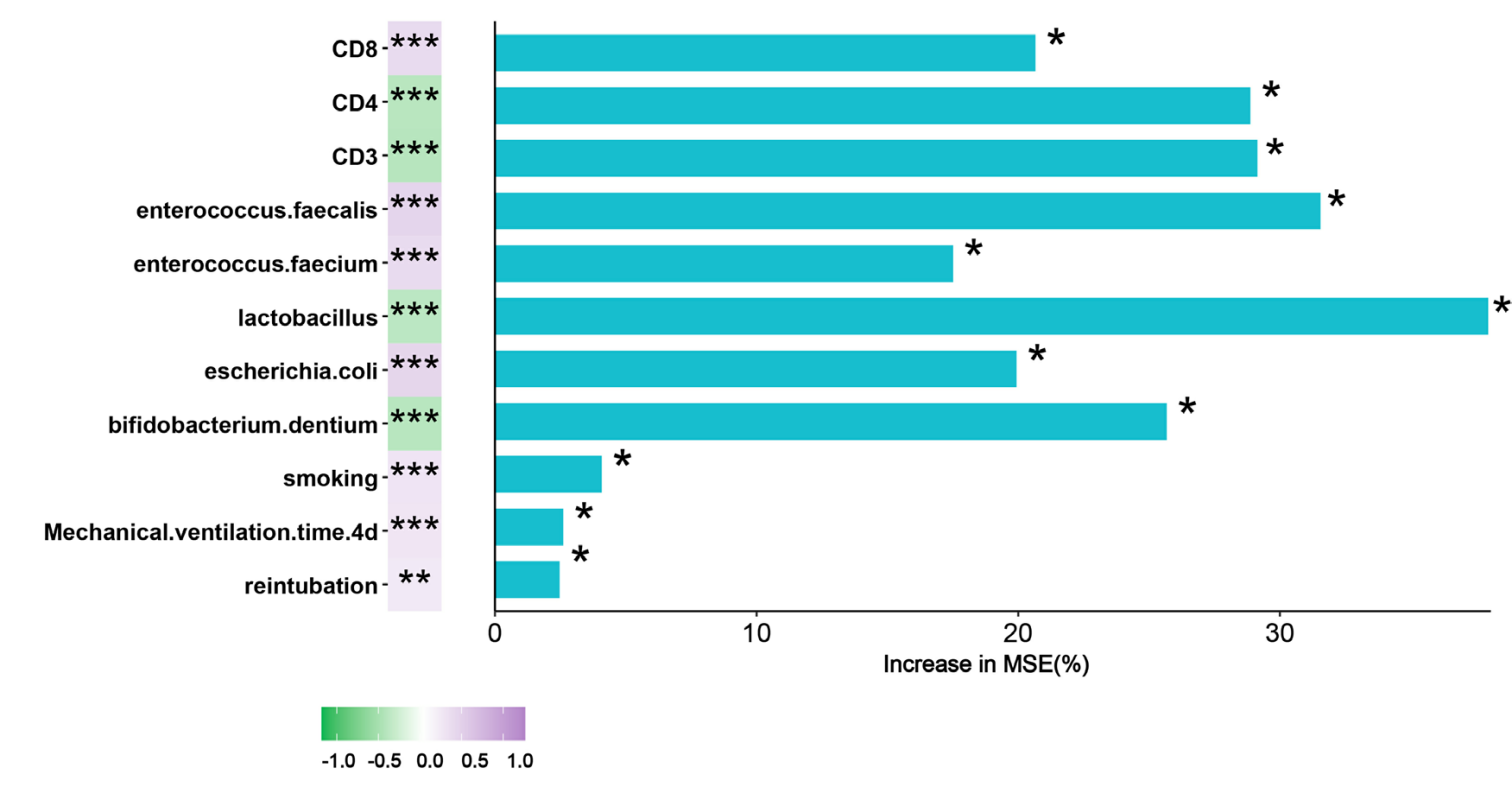
Weights and correlations of factors affecting VAP in COPD patients. COPD = chronic obstructive pulmonary disease, VAP = ventilator-associated pneumonia.

A random forest model was constructed based on the factors with statistically significant differences in Table 2 (reintubation, mechanical ventilation time ≥ 4 days, smoking, Bifidobacterium, Escherichia coli, Lactobacillus, Enterococcus faecium, Enterococcus faecalis, CD3^+^, CD4^+^, CD8^+^), and the value assignment was consistent with 2.3. As can be seen from Figure 3, feature importance was ranked using the %IncMSE scoring method. The top 3 important features were Lactobacillus, *Enterococcus faecalis*, and CD3^+^, with %IncMse × 10–^2^ being 37.962%, 31.552 %, and 29.141%, respectively. The random forest model *P *= .01, *r^2^ *= 0.538.

**Table 2:** Binary logistic regression analysis of factors affecting the occurrence of VAP in COPD patients.

Influencing factors	*B*	*SE*	Wald	*P*	OR	95% confidence interval
Reintubation	1.030	0.484	4.528	.033	2.800	1.085–7.229
Mechanical ventilation time ≥ 4 days	1.125	0.445	6.385	.012	3.079	1.287–7.366
Smoking	1.540	0.474	10.560	.001	4.665	1.843–11.809
*Bifidobacterium*	−0.584	0.162	12.960	<.001	0.557	0.406–0.766
*Escherichia coli*	0.577	0.214	7.280	.007	1.781	1.171–2.707
*Lactobacillus*	−0.221	0.098	5.055	.025	0.801	0.661–0.972
*Enterococcus faecium*	0.294	0.100	8.684	.003	1.342	1.104–1.633
*Enterococcus faecalis*	0.470	0.116	16.488	<.001	1.600	1.275–2.008
CD3^+^	−0.627	0.274	16.549	<.001	0.534	0.395–0.722
CD4^+^	−1.053	0.274	14.791	<.001	0.349	0.204–0.597
CD8^+^	−2.007	0.347	33.402	<.001	0.134	0.068–0.265
FVC	−0.959	0.677	2.010	.156	0.383	0.102–1.443
TNF-α	0.004	0.003	1.735	.188	1.004	0.998–1.011
CAT	0.027	0.050	0.303	.582	1.028	0.933–1.132
Constant	98.959	17.674	31.349	<.001	-	-

CAT = COPD assessment test, FVC = forced vital capacity, TNF-α = tumor necrosis factor-α.

### 3.6. The value of random forest model in predicting VAP in COPD patients

**Figure 4.**
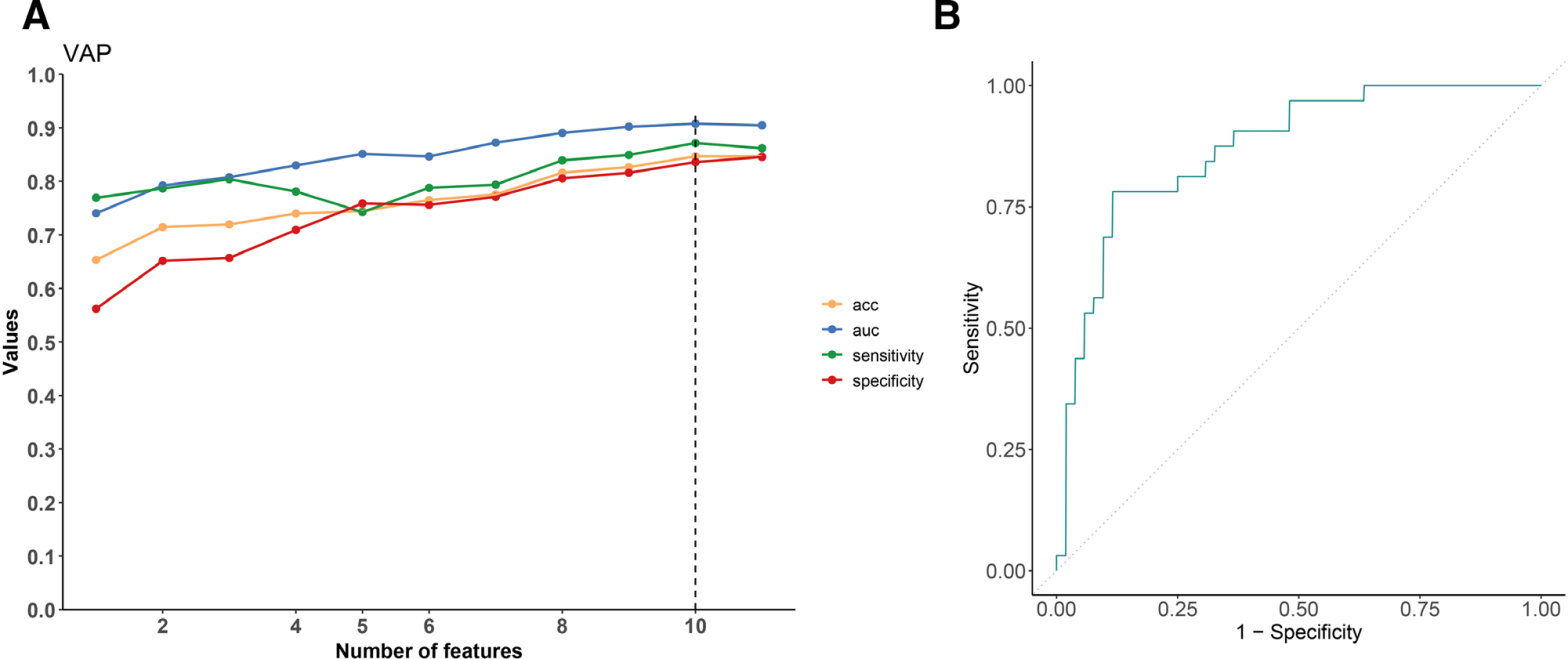
The value of random forest model in predicting the occurrence of VAP in COPD patients. (A) Random forest model performance for VAP diagnosis using 10 key clinical and microbial factors (AUC = 0.908). (B) Random forest model performance for VAP prediction in COPD patients (AUC = 0.857). AUC = area under the curve, COPD = chronic obstructive pulmonary disease, VAP = ventilator-associated pneumonia.

As shown in Figure 4, the random forest model was constructed using the random Forest package in R (R4.1.0) and was internally validated using Bootstrap resampling (with 500 iterations). The validation results demonstrated that the model achieved optimal diagnostic performance when incorporating 10 key factors (reintubation, mechanical ventilation time ≥ 4 days, *Bifidobacterium, Escherichia coli, Lactobacillus, Enterococcus faecium, Enterococcus faecalis*, CD3^+^, CD4^+^, and CD8^+^). The AUC was 0.908, with an accuracy of 0.847, a sensitivity of 0.871, and a specificity of 0.836. Internal validation further revealed a mean AUC of 0.909 (95% confidence interval [CI]: 0.848–0.955), a mean accuracy of 0.832 (95% CI: 0.762–0.895), a mean sensitivity of 0.792 (95% CI: 0.666–0.923), and a mean specificity of 0.866 (95% CI: 0.779–0.941).

## 4. Discussion

VAP is a common hospital-acquired pneumonia and one of the most common complications of mechanical ventilation.^[12]^ Its development results from a combination of pathogenic microorganisms and various contributing factors.^[13]^ Despite preventive measures and antibiotic therapies, VAP remains a persistent clinical challenge. Studies have shown that VAP increases the average hospital stay of mechanically ventilated patients by 7 to 10 days, with associated mortality rates reaching 15.5% to 38.2%.^[14]^ In a survey conducted in mainland China, the reported VAP incidence ranged from 20% to 71%, while this study observed a rate of 44.29%, indicating a relatively high occurrence within the expected range^[15]^ Therefore, identifying VAP risk factors in COPD patients and implementing targeted interventions is crucial to reduce its incidence and improve patient outcomes.

Current clinical studies have found that the pathogens of VAP infection are mainly Gram-negative bacteria, including Escherichia coli and Pseudomonas aeruginosa, and the sources of VAP infection can be divided into 2 aspects: exogenous and endogenous.^[16]^ Among them, the exogenous source mainly comes from the hospital environment, such as poor hand hygiene of medical staff, unclean respiratory equipment and poor infection between patients.^[17]^ Studies have shown that about 20% of medical staff have common Gram-negative bacteria colonization on their hands. Additionally, many retrospective studies have shown that effective hand hygiene can significantly reduce the incidence of VAP in patients by more than 50%.^[18,19]^ However, in addition to exogenous infection, endogenous potential pathogens also play an important role in the occurrence of VAP. The gastrointestinal tract and oral periodontal tissues are considered potential reservoirs of pathogenic bacteria. The intestinal flora is the largest and most complex microecological system in the human body. According to their effects on the human body, they can be divided into beneficial bacteria, harmful bacteria and neutral bacteria. Beneficial bacteria such as Bifidobacterium and Lactobacillus can participate in food digestion, promote intestinal peristalsis, and inhibit the proliferation of pathogenic bacteria.^[20]^ Harmful bacteria such as Escherichia coli and Enterococcus faecium can induce gastrointestinal infections and functional disorders. If they are displaced and abnormally colonized, they can induce extraintestinal infections.^[21]^ Studies have confirmed through mouse experiments that an imbalance in intestinal flora will further aggravate the severity of Mycoplasma pneumoniae respiratory tract infections.^[22]^ This shows that the intestinal flora may be involved in the occurrence and development of pneumonia. Based on the previous study, in addition to finding that changes in the intestinal flora of COPD patients are related to the progression of the patient’s disease, this study also found that *Bifidobacterium, Escherichia coli, Lactobacillus, Enterococcus faecium*, and *Enterococcus faecalis* are related to changes in the levels of T lymphocyte subsets in patients. Intestinal flora may participate in the occurrence of VAP by affecting the levels of T lymphocyte subsets in patients and regulating the body’s immune function. Understanding the precise role of intestinal flora could pave the way for new intervention strategies targeting COPD-associated VAP, its mechanism of action needs to be deeply understood. However, this study assessed the fecal microbiota using traditional culture methods, which presents certain limitations. This approach can only detect culturable microorganisms (estimated to represent only 1–30% of the total gut microbiota), potentially missing a large number of strict anaerobes (e.g., *Bacteroides* and *Prevotella*) and difficult-to-culture species, leading to a significant underestimation of microbial diversity. Furthermore, culture results are highly dependent on technical parameters such as the culture medium type, oxygen conditions, and incubation duration, introducing a risk of selection bias. Future studies could incorporate multidimensional validation using techniques such as 16S rRNA gene sequencing or metagenomic sequencing to more comprehensively elucidate the potential relationship between the gut microbiota and VAP in patients undergoing noninvasive ventilation.

As clinical research into the mechanisms of hospital-acquired pneumonia infection deepens, immune dysfunction is increasingly recognized as a critical factor contributing to pathogen invasion and subsequent infection.^[23]^ T lymphocytes are key components of the immune system, and a study by Qin K^[24]^ indicated that in COPD patients, as the disease progresses, T lymphocyte-mediated immune regulation is suppressed, leading to immune dysfunction and an increased risk of infection. As an important component of human immunity, the intestine participates in the regulation of the immune function of the body and distant organs. Among them, the gut-associated lymphoid tissue (GALT) plays an important role in maintaining lung immune regulation.^[25]^ GALT can be divided into the epithelial layer and the lamina propria. The lamina propria can participate in the regulation of the body’s T lymphocytes and plays an important role in promoting the conversion of CD8^+^ to CD4^+^ and maintaining the homeostasis of helper T cells 17/regulatory T cells.^[26]^ Additionally, in the study of Lv M,^[27]^ it was found that intestinal Gram-negative bacterial lipopolysaccharide and flagellin have immune activation effects. When the immune system senses flagellin, it can specifically produce activating antimicrobial peptides and chemokines to activate T lymphocytes to produce immune responses. Hufnagl K^[28]^et al suggested that the lungs and intestines can achieve 2-way communication through the blood system, thereby controlling the patient’s lung immune activation and participating in the occurrence and development of respiratory diseases. This shows that the intestinal flora of COPD patients can affect the body’s immune function in multiple dimensions, thereby participating in the body’s infection process.

As the “second genome” of the human body, the intestinal flora has been found to play an important role in the pathophysiological process of many diseases.^[28,29]^ Therefore, this study analyzed the differences in intestinal flora between VAP and non-VAP patients. The results showed that the levels of bifidobacteria and lactobacilli in non-VAP patients were lower than those in non-VAP patients, while those of Escherichia coli, Enterococcus faecium, and Enterococcus faecalis were higher than those in non-AVP patients, indicating that VAP patients had a higher proportion of harmful intestinal bacteria and more serious intestinal flora disorders. Additionally, this study further constructed a regression model and found that Escherichia coli, Enterococcus faecium, and Enterococcus faecalis were risk factors for VAP in COPD patients, while bifidobacteria and lactobacilli were protective factors, indicating that the intestinal flora of COPD patients was involved in the occurrence of VAP. According to relevant studies, lactobacilli can interact with dendritic cells, affect T cell differentiation, and inhibit the production of pro-inflammatory factors (tumor necrosis factor-α) by monocytes, thereby participating in the body’s immune regulation.^[30]^ Lactobacilli can directly damage pathogens by synthesizing hydrogen peroxide to cause oxidative damage to pathogenic bacteria DNA and proteins, thereby reducing the risk of pathogen infection in patients.^[31]^ Bifidobacteria can promote the expression of tight junction proteins and inhibit the activation of Toll-like receptor 4, thereby reducing the body’s inflammatory response and protecting the intestinal barrier function by restoring intestinal permeability and cellular levels.^[32]^ Bifidobacteria are the only nonpathogenic strains that do not produce endotoxins and exotoxins. The bifidobacterial peptides they secrete have a wide range of antibacterial effects and can inhibit conditional pathogens in the intestine. Additionally, bifidobacteria can also secrete short-chain fatty acids to lower the intestinal pH value, inhibit the colonization and proliferation of facultative anaerobic bacteria and foreign bacteria in the intestine, thereby reducing the risk of.^[33]^ Therefore, when the levels of lactobacilli and bifidobacteria decrease, the intestinal mucosal biological barrier function is affected, resulting in the opportunity for pathogens to pass through the intestinal mucosa to the lamina propria, and then reach the mesenteric lymph nodes or even further, releasing inflammatory transmitters and bioactive factors, thereby increasing the risk of VAP.^[34]^ Escherichia coli, Enterococcus faecium, and Enterococcus faecalis are all common opportunistic pathogens in clinical practice. When they proliferate in large numbers, they can secrete and release large amounts of enteric endotoxins, which directly cause lung damage through the inferior vena cava, right atrium, pulmonary artery, and capillaries, aggravating the condition of COPD patients, prolonging ventilation time, and thus increasing the risk of VAP.^[35]^ The above analysis explains the physiological role of Bifidobacterium, Lactobacillus, Escherichia coli, Enterococcus faecium, and Enterococcus faecalis in the occurrence of VAP in COPD patients. However, in order to better apply the results of this study to clinical practice, further analysis of the value of intestinal flora in prediction is needed.

In order to fully understand the value of intestinal flora in predicting the occurrence of VAP in COPD patients, this study constructed a random forest model to evaluate the risk of VAP in COPD patients. The results showed that the forest model predicted the occurrence of VAP in COPD patients with an AUC of 0.857, an accuracy of 0.786, a sensitivity of 0.778, and a specificity of 0.7921, indicating that the intestinal flora has a high efficiency in predicting the risk of VAP in COPD patients and has clinical application value. In the future, clinical practice can evaluate the characteristics of intestinal flora in COPD patients and adopt personalized intervention plans for high-risk patients to help patients restore the balance of intestinal flora, thereby reducing the risk of VAP and improving patient prognosis. However, this study has some limitations. First, the sample size is relatively small, and it is a single-center study, which may affect the generalizability of the results. Moreover, the study did not observe the changes in microbiota levels during the patient’s treatment period, only assessing the microbiota at admission. Additionally, this study only measured the gut microbiota at the time of admission, without monitoring its dynamic changes throughout the treatment period. Observing the relationship between shifts in the gut microbiota and the occurrence of VAP, as well as patient prognosis, could potentially facilitate the development of more informed clinical strategies. Future research should aim to increase the sample size by extending the study duration and incorporating a multi-center design, while also conducting repeated measurements of the gut microbiota. The approach would allow for a more in-depth investigation into the relationship between the gut microbiota and the occurrence and prognosis of VAP in this patient population.

## 5. Conclusion

In conclusion, this study preliminarily suggests that the gut microbiota characteristics of COPD patients treated with noninvasive ventilator may be associated with immune function and could be involved in the pathogenesis of VAP. The random forest model based on intestinal flora demonstrated preliminary predictive value for VAP occurrence; however, larger-scale, multicenter studies are warranted to further validate its clinical applicability.

## Acknowledgments

The authors have no acknowledgments to report.

## Author contributions

**Conceptualization:** Yanbo Liu.

**Data curation:** Yanbo Liu, Chengjiang Liu, Caifeng Huang, Lisha Pang.

**Formal analysis:** Chengjiang Liu.

**Investigation:** Yanbo Liu, Chengjiang Liu, Caifeng Huang, Lisha Pang.

**Methodology:** Yanbo Liu, Chengjiang Liu, Caifeng Huang, Lisha Pang.

**Project administration:** Lisha Pang.

**Resources:** Chengjiang Liu, Caifeng Huang, Lisha Pang.

**Software:** Yanbo Liu, Caifeng Huang.

**Supervision:** Yanbo Liu, Chengjiang Liu.

**Validation:** Yanbo Liu, Lisha Pang.

**Visualization:** Yanbo Liu, Chengjiang Liu, Caifeng Huang.

**Writing – original draft:** Chengjiang Liu.

**Writing – review & editing:** Caifeng Huang, Lisha Pang.
